# Assessing the Mediation Mechanism of Job Satisfaction and Organizational Commitment on Innovative Behavior: The Perspective of Psychological Capital

**DOI:** 10.3389/fpsyg.2019.02699

**Published:** 2019-12-17

**Authors:** Yuan Tang, Yun-Fei Shao, Yi-Jun Chen

**Affiliations:** ^1^Institute of Management, Sichuan University of Science and Engineering, Zigong, China; ^2^School of Management and Economics, University of Electronic Science and Technology of China, Chengdu, China

**Keywords:** psychological capital, job satisfaction, organizational commitment, employee innovative behavior, partial least squares

## Abstract

Due to increasingly intense competition among companies, employees’ innovative behavior has not only become a crucial factor for company development but also a topic of broad and current interest among companies and researchers. It is a requisite for companies to identify the antecedents of employees’ innovative behavior. The main objective of this study was to investigate the effect of psychological capital (PsyCap) on employees’ innovative behavior through its relationship with job satisfaction and organizational commitment. The partial least squares method was adopted in this study to analyze 266 employees from China. The results showed that PsyCap had positive effects on job satisfaction and organizational commitment, and verified the relationship between employees’ innovative behavior and their job satisfaction and organizational commitment. Moreover, the mediating effect of PsyCap in terms of job satisfaction and organizational commitment on employees’ innovative behavior was verified by a mediation analysis. Employees’ innovative behavior is not only essential for the research and development department; rather, it is also important for other departments. The empirical results of this study show that companies should consider taking measures to increase employees’ PsyCap, so as to enhance their innovative behavior. Lastly, the study also provided the managerial implications of its findings and recommendations for future research.

## Introduction

Due to the rapid transformation of the global economy, companies nowadays have to face complex and ever-changing competitive environments. Leaders and members of an organization must be able to adapt to rapid changes in the working environment in order for the organization to pursue sustainable development (Spreitzer and Porath, 2012; Spreitzer et al., 2012). In such a highly uncertain environment, management costs can be saved and organizational effectiveness can be enhanced if employees are able to proactively seek the information required to achieve their work objectives and, thereby, improve their work performance (Shipton et al., 2006; Grant and Ashford, 2008). Employees’ innovative work behavior has always been an important factor for organizations to innovate and increase their competitiveness. Employees’ expression of creativity in the workplace is beneficial for the innovation of the organizations’ products, services, and workflows. For instance, knowledge workers are valuable human assets for companies in the knowledge-intensive high-tech industry (Fritz et al., 2011). As China shifts from a manufacturing giant to a knowledge-based economy, employees in knowledge-based companies have slowly transformed into working groups whose main job is to create, apply, and increase knowledge. These employees have a passion for challenging and creative tasks while striving to achieve perfection. Through the process of completing these tasks, they aspire to fully express their attributes, fulfill their self-worth, and gain approval and respect from organizations or teams. Therefore, it is important for companies to identify the important factors that promote employees to express their unparalleled creativity in the innovation of new products or services, so as to keep up with business trends and enhance innovative performance (Carmeli and Spreitzer, 2009).

Psychological capital (PsyCap) is reflective of a worker’s positive mental energy and is an important intangible capital for companies (Avey et al., 2011; Baron et al., 2016; Manuti and Giancaspro, 2019). PsyCap can be effectively enhanced through specific development and training processes, such that it becomes an important capital for organizations (Luthans et al., 2005). PsyCap is an individual’s positive psychological state of mind, and is characterized by self-efficacy, hope, optimism, and resilience (Luthans et al., 2005). In 2000, while acting as the president of the American Psychology Association, Seligman and Csikszentmihalyi (2000) proposed his theory of positive psychology, which was subsequently explored by many researchers. In this context, research on happiness has received increasing attention in the field of psychology. The field of positive psychology emphasizes the development of an individual’s strengths, so as to promote their positive functioning, such as the characteristics or abilities that allow the individual to lead a better and happier life. Luthans et al. (2007a) proposed the concepts of positive PsyCap and defined positive PsyCap as the core construct of an individual’s positive psychological state, which refers to the individual’s confidence (or self-efficacy), hope, optimism, and resilience. In addition, they suggested that positive PsyCap can help an individual to adapt to their environment and stress, develop their competitive advantage, and improve their well-being.

Previous research have suggested that the support and encouragement an individual receives have positive impacts on their performance (Seligman and Csikszentmihalyi, 2000). Concepts of positive psychology, such as subjective well-being, positive PsyCap, and role identity, describe the abilities, attitudes, and values of workers, which are crucial for a company’s innovations. Consider the following scenarios: (i) If an optimistic IT worker encounters a problem, would they have the hope and confidence to solve the problem? (ii) If an optimistic IT worker finds themselves facing adversity, would their psychological state be easily affected by the harsh conditions they face? (iii) If an IT worker has a high sense of identification with their role, are they able to bravely accept challenges while implementing a project to develop new systems? Seligman (1988) and Luthans et al. (2007a) have pointed out that if an individual has been able to proactively develop the characteristics of positive PsyCap, they would have the confidence to achieve success in challenging tasks, in addition to having optimistic views on problems encountered in the present or future. Meanwhile, the individual would also attempt various approaches to achieve their goals, and would persevere even if they had failed. These studies demonstrated that an individual’s optimism is positively related to their work performance or learning outcomes.

Advancements in technology, increasingly intense competition among companies, and innovation in products and technology can easily cause imitative behavior among competitors. Hence, organizations and companies should consider measures that can strengthen employees’ innovative behavior. Based on the perspectives of positive psychology, this study aimed to investigate how employees’ PsyCap affects their job satisfaction and their organizational commitment, thereby increasing their corporate coherence and innovative behavior. The empirical results of this study are expected to be beneficial for enhancing the managerial implications and the development of a company’s academic and practical innovations.

## Theoretical Background and Literature Review

### Psychological Capital

In regard to the research on positive organizational behavior, Luthans et al. (2007a) believed that while there was no lack of research on personal positive constructs (such as self-efficacy and positivity) in the field of organizational behavior, a higher-level construct with a broader scope should be developed and used to represent an individual’s psychological capacity. Based on previous literature and relevant theoretical derivations, Luthans et al. (2007b) proposed the concept of PsyCap and its four constructs, which are self-efficacy, optimism, resilience, and hope. Self-efficacy is a core principle in social cognitive theory, and is also one of the most often discussed concepts in behavioral science (Stajkovic and Luthans, 1998). Self-efficacy refers to an individual’s belief in their capabilities to complete tasks. This belief also has positive predictive power on an individual’s learning outcomes, interpersonal relationships, and work performance (Zimmerman, 2000; Judge et al., 2007). If an individual believes that they are capable of facing challenges, not only are they able to effectively de-stress, they are also able to adaptively rise up following setbacks in the workplace. This mentality is also beneficial for overcoming possible challenges during the process of innovation (Schaubroeck and Merritt, 1997; Tierney and Farmer, 2002).

Hope is rooted in hope theory (Snyder, 2002), and its essence covers motivation, direction, and goals. In other words, hope is an individual’s belief in their determination to achieve their goals and to find possible pathways to overcome the difficulties that they encounter. Optimism is based on the clear appraisal and judgment of current situations, and on understanding what can be done in such situations. This belief can enhance an individual’s self-efficacy and their hope for a better future (Fredrickson, 2004). Regarding the difference between hope and optimism, the annotations of Snyder (2002) stated that hope is a motivational state with direction, while optimism focuses on having outlooks and making self-adjustments according to different circumstances. In previous empirical studies, both constructs showed distinct and significant predictive powers (Bailey et al., 2007). Resilience refers to the ability to recover from adversity or setbacks, proactively rise up to challenges, and adapt to an ever-changing business environment. An individual’s resilience is beneficial for them in terms of seeking opportunities and strengthening their job-seeking skills (Fleig-Palmer et al., 2009), as well as making flexible adjustments when facing adversity in order to achieve desirable performance (Inzlicht et al., 2006). In other words, resilience is not only about enduring hardships in a passive manner, but also about seeking out opportunities to improve one’s situation (Luthans et al., 2007a). Based on the literature available, it can be seen that there are significant correlations between job satisfaction and hope, optimism, resilience, and work performance (Youssef and Luthans, 2007).

### Job Satisfaction

Job satisfaction refers to the feelings or affective responses of a worker regarding factors such as the job itself, work experience, and the working environment (Robbins et al., 2015). It is the general attitude of workers’ satisfaction or dissatisfaction with their jobs. A worker may feel more positive about their work if they are satisfied. The main objective of job satisfaction is to understand the current requirements of employees, and even though it is not the only factor determining the behavior of organizational members, it is a crucial factor affecting their behavior. An in-depth investigation of job satisfaction can help companies perform organizational diagnoses that in turn help them to improve their current operations and management. A study by Konstantinos and Zampetakis (2008) indicated that positive and negative work factors had a mediating effect between emotional control and job satisfaction. This effect was more pronounced in males as they were more influenced by positive work factors. Therefore, researchers have always been interested in the relationship between job satisfaction and work performance.

The theoretical frameworks of studies differ according to their perspectives and objectives. Hence, job satisfaction can be defined through different concepts, and these concepts are often categorized as follows:

• Overall satisfaction is defined as a unitary concept in which workers are able to balance the satisfaction and dissatisfaction from different job dimensions and achieve overall satisfaction with their jobs. This concept emphasizes the attitude of workers toward their working environments, and is the process of psychological change in a worker’s personal satisfaction with their job. It does not involve the facets, causes, and process of the formation of job satisfaction.
• Expectation discrepancy is defined as the gap between an individual’s expected value and the actual value that they receive in a specific working environment. In other words, based on this discrepancy, job satisfaction can be defined as the gap between the actual value an individual receives from their working environment and their expected reward. Robbins (2005) suggested this approach for measuring job satisfaction. However, this approach negates the degree of satisfaction that the job itself presents to workers and emphasizes the workers’ satisfaction. Job satisfaction is determined by the gap between the expected and perceived actual values. Satisfaction is low when the gap is large, yet it is difficult to measure this gap.
• In the frame of reference concept, employees will explain and compare their job characteristics based on factors such as job facets, personal reasons, and the job itself. This concept emphasizes a worker’s affective response to characteristics of their job. The most important factor that influences the attitudes and behaviors of workers is the subjective perceptions and interpretations a worker has regarding their job characteristics, and not the objective evidence within the job or organization. Common dimensions include remuneration, working environment, and working groups. Many studies have used the Minnesota Satisfaction Questionnaire, in which job satisfaction consists of intrinsic and extrinsic satisfaction (Karsh et al., 2005).

Based on the objectives of this study and the perspectives of the aforementioned literature, job satisfaction is defined in this study as “the perceived agreement of an employee’s expectations with their actual work process, responsibilities, and job context.” Job satisfaction is high if most of their expectations are met, and vice versa. Since the concept of job satisfaction is broad, the unitary concept of overall satisfaction, which refers to the overall feeling or affective response of an employee regarding their role, was used in this study. Therefore, the attitudes or perspectives of employees on their job and working environment were emphasized in this study. The multidimensional analysis of job satisfaction, as well as the cause and process of the formation of job satisfaction, was not considered in this study.

### Organizational Commitment

Organizational commitment is an internalized normative force that enables the integration of behavior with organizational goals and interests. However, scholars have inconsistent definitions for organizational commitment, as these definitions differ according to the variety of schools of thought and research backgrounds and objectives. Alpander (1990) defined organizational commitment as an organizational member’s sense of loyalty or affection toward their attachment to their organization. Organizational commitment is an important topic in the research of organizational behavior, as employees with high organizational commitment are able to identify with the organizational goals and values, and are willing to exert extra effort in completing their work.

Organizational commitment refers to an individual’s degree of identifying and engaging with a specific organization, which enables organizational members to internalize organizational goals and express behaviors that are beneficial for the organization (Mowday et al., 1982). The degree of organizational commitment is positively related to the degree of expression of employees’ organizational citizenship behavior. Podsakoff et al. (2000) suggested that employees expressed organizational citizenship behavior as a form of supportive feedback for their organization. Organizational commitment refers to the relationship between employees and their organizations. Mowday et al. (1982) pointed out that organizational commitment includes the identification of employees with organizational goals and values, and their devotion, engagement, loyalty, willingness to maintain membership, and expression of proactive behavior toward their organizations. Meyer and Allen (1991) conceptualized organizational commitment into three components: (i) affective commitment refers to the belief in and acceptance of organizational goals and values; (ii) continuance commitment refers to the perceived loss in values and benefits when an employee leaves their organization; and (iii) normative commitment refers to the consistency between individual and organizational values, or the obligation to maintain in the organization due to work responsibilities. Organizational commitment is an important factor for understanding employees’ work behavior, as it constitutes an attitude or orientation toward the organization which links or attaches the individual to the organization. Therefore, organizational commitment can be viewed as a behavior or a set of behavioral intentions and attitudes, which has a certain degree of effect on organizational members’ behavioral outcomes (Goulet and Frank, 2002).

When employees identify with their organization and its goals, they will want to become a part of the organization, and organizational commitment will become negatively correlated to employee absenteeism and turnover rates (Robbins, 2005), which shows that organizational commitment is an emotional expression of an individual’s sense of belonging, identification, and participation (McShane and Von Glinow, 2003). In other words, members with high organizational commitment are capable of increasing organizational coherence and competitiveness, and vice versa, that is, members with low organizational commitment feel insecure toward their organization or have turnover intentions. Individuals and organizations share an interdependent relationship, and organizational commitment is not determined solely by either party. Therefore, organizations should take measures to strengthen employees’ sense of responsibility and enhance or develop proactive job attitudes, thereby strengthening employees’ identification and attachment to their organizations to increase and enhance their beliefs in the organizations and their goals. Companies should recruit talents whose personal values and beliefs are consistent with organizational values. Organizations should encourage employees to express proactive actions and attitudes to enhance their trust toward the organization, so as to build desirable working environments within the organization and stimulate employees’ work motivation and job satisfaction. On the other hand, employees’ job attitudes are crucial, as employees’ loyalty and determination toward their jobs is important for organizations to realize their goals. Employees’ expression of organizational commitment also indicates that they wish to maintain in and work for the organization.

Based on the aforementioned literature, organizational commitment is defined in this study as an organizational member’s strong acceptance of organizational goals and values, as well as their willingness to devote themselves to the organization and maintain their position.

### Employee Innovative Behavior

According to the mantra “innovation or die,” innovation is the main source of an organization’s competitive advantage (Drucker, 1999). Organizational innovation originates from the expression of innovative behavior in members toward their jobs, which includes the use of creativity, sensitivity in problem discovery, and taking advantage of opportunities to evoke proactive creative thinking and implement creative ideas to develop new products, services, or even create new markets. Therefore, organizational innovation researchers are always delving into approaches to evoke the creativity of organizational members or to encourage them to implement their creative ideas (Scott and Bruce, 1994; Yuan and Woodman, 2010; Anderson et al., 2014). This also shows that high-tech companies like Apple, Google, and Facebook are always implementing various management practices to shape pleasant and comfortable working environments, so as to stimulate employees’ innovative motivation or enthusiasm and to attract top talents.

Innovative behavior refers to the process of developing, finding support, and implementing new ideas (Scott and Bruce, 1994), or the development, introduction, and application of new ideas within responsibilities, working groups, or organizations (Janssen, 2000). Employees’ innovative ideas are crucial for organizations, as they increase job efficiency and enhance organizational performance (Baer and Frese, 2003). Therefore, many studies strive to seek approaches to stimulate organizational employees’ innovative behavior and create desirable working environments, as well as the practicality of supporting and assisting employees to implement their innovative ideas (Bandura, 1986; Tierney and Farmer, 2002).

The effect of organizational social context on members’ innovative behavior can be reflected through the members’ process of self-cognition (Yuan and Woodman, 2010). Therefore, employees must not only be able to feel the organization’s management practices to support innovation, but must also believe that they are capable of achieving innovation tasks. The employees’ self-confidence or creative self-efficacy in completing innovation tasks is an important factor for motivating individuals to do their best to achieve these tasks (Tierney and Farmer, 2002). The employees’ process of sense-making and self-determination is mediated by creative self-efficacy, that is, the employees’ agency with respect to innovation tasks.

Employees’ innovative behavior is beneficial for companies to develop novel and useful ideas and solutions for relevant products, services, processes, and procedures. Nowadays, companies must face rapid changes in technology and harsh business environments. Employees’ innovative behavior is defined in this study as the overall performance of an employee in the process of creative searching, establishing, implementing, and successful realizing of new technologies, processes, techniques, or products, so as to generate useful products or services.

## Research Method

### Hypotheses Development

The empirical results of a study by Luthans et al. (2007a) on the relationship between PsyCap, job efficiency, and job satisfaction supported the positive effects of PsyCap on job satisfaction. Judge et al. (2001) suggested that the confidence (self-efficacy) construct in PsyCap influences the degree of job satisfaction, while Peterson and Luthans (2003) and Luthans and Youssef (2004) agreed that the degree of hope influences the job satisfaction and work performance of managers and employees. Recent studies have also supported the relationship between PsyCap and job satisfaction or performance (e.g., Luthans et al., 2007b; Avey et al., 2008; Walumbwa et al., 2009). A summary of the aforementioned literature suggests that the level of PsyCap of professionals and workers, regardless of career fields, is positively related with their job satisfaction, i.e., the better the PsyCap, the higher the job satisfaction. By increasing their PsyCap, employees may be able to set attainable work objectives and are less likely to back down from setbacks. They will have stronger motivation to face difficulties at work while controlling their stress and anxiety, devote themselves to solving problems while being continuously hardworking, and will not give up easily or feel helpless, thereby increasing their job efficiency and overall job satisfaction. Based on the discussions above, the following hypothesis (H1) was derived in this study:

Hypothesis 1: Psychological capital positively influences job satisfaction.

An empirical study on the hotel workplace by Wu and Chen (2018) revealed that PsyCap had positive effects on organizational commitment. In the medical and healthcare industry, a study by Zhou et al. (2018) underlined that the PsyCap of nurses with high stress and high workload was positively related to their organizational commitment. Another study by Çetin (2011) pointed out that the PsyCap constructs of hope and optimism of 213 employees in Turkey had significant effects on their job satisfaction and organizational commitment. Other relevant studies have also shown similar causal effects (e.g., Zhong, 2007; Newman et al., 2018). Hence, the following hypothesis (H2) was derived in this study:

Hypothesis 2: Psychological capital positively influences organizational commitment.

A study by Schwepker (2001) on the ethical climate of salespeople revealed that organizational commitment was the mediator between their job satisfaction and turnover intention. According to relevant studies, there is significant positive correlation between job satisfaction and organizational commitment (Chu et al., 2005; Kim et al., 2005; Macintosh and Krush, 2014; Veličković et al., 2014), and the following hypothesis (H3) was derived:

Hypothesis 3: Job satisfaction positively influences organizational commitment.

In an organization, an employee’s satisfaction with their job often affects their degree of work engagement and the degree of relevance with organizational goals. An employee with a high job satisfaction has less turnover intentions (Poon, 2003), which is beneficial for the development of the organization (Reiner and Zhao, 1999). Innovative measures should be taken by traditional and high-paying companies to improve their competitiveness and enhance their performance, as innovation has positive effects on company performance (Damanpour et al., 1989; Khan and Manopichetwattana, 1989; Luoh et al., 2014). Therefore, the following hypothesis was derived in this study:

Hypothesis 4: Job satisfaction positively influences innovative behavior of employees.

Organizational commitment refers to an individual’s willingness to devote and be loyal to an organization. Therefore, organizational commitment is an internalized normative force that promotes the organization members’ willingness to conform to the organizational goals and interests. Employees will agree with organizational goals and values if they have a strong sense of organizational commitment, and are more willing to express extra-role behaviors. Mathieu and Zajac (1990) also proposed that an individual’s innovative behavior is an expression of their extra role behavior. Based on literature, there are positive relationships between organizational commitment and employees’ innovative behavior (Jafri, 2010). Summarizing the discussions above, the following hypothesis was derived in this study:

Hypothesis 5: Organizational commitment positively influences innovative behavior of employees.

**FIGURE 1:**
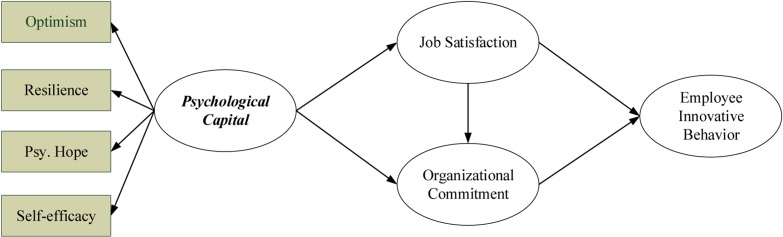
Research model.

For the research model framework of this study, PsyCap was selected as the basis, and its effect in on employees’ innovative behavior via job satisfaction and organizational commitment was investigated. The research model and research hypotheses are shown in Figure 1 and Table 1, respectively.

**TABLE 1:** Research hypotheses.

Hypothesis 1	Psychological capital positively influences job satisfaction.
Hypothesis 2	Psychological capital positively influences organizational commitment.
Hypothesis 3	Job satisfaction positively influences organizational commitment.
Hypothesis 4	Job satisfaction positively influences innovative behavior.
Hypothesis 5	Organizational commitment positively influences innovative behavior.

### Measurement Items and Sample Structure

In this study, PsyCap was measured using the questionnaire developed by Luthans et al. (2007b), which consists of the four constructs of PsyCap, i.e., self-efficacy (six items), optimism (six items), resilience (five items), and hope (five items). Items developed by Kiffin-Petersen and Cordery (2003) were modified for measuring job satisfaction. Six items developed by Meyer and Allen (1991) were chosen for measuring organizational commitment, and six items formulated by Tsai and Kao (2004) and Scott and Bruce (1994) were used to measure employees’ innovative behavior. The questionnaire was answered on a seven-point Likert scale (1 = strongly disagree, 7 = strongly agree). The variables, items, and relevant references within the research model framework for this study are summarized in Table 2.

**TABLE 2:** Operational definition of constructs.

**Construct**	**Definition**	**Source**
Psychology capital	Psychology capital is characterized by confidence, optimism, hope, and resilience.	Luthans et al., 2007b
Job satisfaction	The general attitude of an individual toward their job. It represents the degree of satisfaction or dissatisfaction of individuals toward their jobs. Employees reflect their feelings by expressing satisfaction and positivity on their jobs and organizations.	Edwards and Rothbard, 1999; Crossman and Abou-Zaki, 2003
Organizational commitment	The degree of employees’ state of being wholeheartedly in approval with the company, and their willingness to stay with the company based on a sense of belonging and happiness, instead of choosing to stay because of continuous commitment or normative commitment.	Meyer and Allen, 1991
Employee innovative behavior	The overall performance of an organizational member in the process of creative searching, establishing, implementing, and successful realizing of new technologies, processes, techniques, or products, so as to generate useful products or services.	Scott and Bruce, 1994

In this study, employees were drawn, respectively, from various departments and from various organizations of different industries. Statistical hypothesis testing was performed on 266 valid samples to increase the external validity of the analysis results. Of the 266 individuals analyzed, 65.8% of the respondents were male, while 34.2% were female. The average of the samples was 32.8 years old. The industry classification of organizations and work roles of employees were not restricted throughout the process of sample collection, as the common behavior of employees from various fields was emphasized in this study, so as to discuss the general implications of different professional fields on employees’ innovative behavior. Table 3 showed the means, standard deviations, and correlations between variables.

**TABLE 3:** Mean, standard deviations, and correlation matrix.

	**Mean**	**SD**	**HOPE**	**OPT**	**RES**	**SEEF**	**SAT**	**ORGC**	**INNO**
HOPE	2.801	1.259	1.000						
OPT	3.071	1.300	0.689	1.000					
RES	2.833	1.322	0.785	0.671	1.000				
SEEF	2.654	1.282	0.708	0.637	0.719	1.000			
SAT	2.952	1.416	0.369	0.438	0.374	0.469	1.000		
ORGC	2.983	1.414	0.405	0.448	0.400	0.507	0.845	1.000	
INNO	2.621	1.275	0.710	0.695	0.696	0.834	0.516	0.579	1.000

*PSY, psychological capital; HOPE, psychological hope; RES, resilience; SEEF, self-efficacy; SAT, job satisfaction; ORGC, Organizational Commitment; INNO, employee innovative behavior.*

## Data Analysis

### Measurement Model

The partial least squares (PLS) regression was adopted as the analytical method in this study mainly because PLS is suitable for investigating the causal effects between construct variables while concurrently processing models that contain construct variables and measurement variables (Petter et al., 2007). Furthermore, as PLS does not necessarily require variables to be normalized or randomized, it can be used to analyze the relationship between variables in a non-normal distribution, in addition to having the ability to analyze complex predictive models (Chin and Newsted, 1999). The main objective of this study was to investigate the causal effects between PsyCap, job satisfaction, organizational commitment, and employees’ innovative behavior, while in literature, each of these constructs consist of various measurement variables. Hence, in order to investigate the causal effects between these variables, reduce measurement errors, and avoid collinearity, PLS was deemed to be more suitable for this study than other analytical methods such as SEM. SmartPLS 3.2.8 analytical tool developed by Ringle et al. (2018) was used in this study.

Regarding the reliability analysis of this study, Cronbach’s alpha and the composite reliability (CR) of potential variables were used to measure the internal consistency of each construct. Reliability is acceptable if the Cronbach’s alpha is 0.7 or greater (Nunnally and Bernstein, 1994). High CR of potential variables indicates strong correlation between construct items and high internal consistency. Fornell and Larcker (1981) recommended that the CR value should be greater than 0.6. As shown in Table 4, the Cronbach’s alpha and CR values of each construct were all greater than the recommended values, which indicates good internal consistency.

**TABLE 4:** Reliability and AVE.

**Construct**	**Cronbach’s alpha**	**Composite reliability**	**Average variance extracted (AVE)**
PSY (second order)	0.938	0.932	0.773
HOPE	0.854	0.892	0.581
RES	0.806	0.866	0.564
SEEF	0.853	0.891	0.579
OPT	0.772	0.868	0.687
SAT	0.887	0.914	0.639
ORGC	0.892	0.917	0.650
INNO	0.883	0.906	0.518

*PSY, psychological capital; HOPE, psychological hope; RES, resilience; SEEF, self-efficacy; SAT, job satisfaction; ORGC, organizational commitment; INNO, employee innovative behavior.*

Convergent validity refers to the degree of correlation between or aggregation of multiple indicators used to measure the same construct. According to Fornell and Larcker (1981) and Hair et al. (2018), convergent validity must fulfill the following criteria: (1) the factor loading of each construct should be greater than 0.7; (2) the CR value should be greater than 0.6; and (3) the average variance extracted (AVE) should be greater than 0.5. As shown in Tables 4, 5, convergent validity exists in this study.

**TABLE 5:** Factor loadings and cross loadings.

	**HOPE**	**OPT**	**RES**	**SEEF**	**SAT**	**ORGC**	**INNO**
HOPE1	**0.764**	0.542	0.628	0.564	0.205	0.237	0.558
HOPE2	**0.808**	0.578	0.617	0.572	0.226	0.265	0.586
HOPE3	**0.669**	0.492	0.568	0.478	0.394	0.365	0.438
HOPE4	**0.703**	0.417	0.516	0.452	0.300	0.305	0.449
HOPE5	**0.819**	0.582	0.627	0.591	0.325	0.381	0.613
HOPE6	**0.797**	0.521	0.626	0.565	0.257	0.306	0.578
OPT1	0.574	**0.829**	0.551	0.488	0.298	0.341	0.552
OPT2	0.554	**0.834**	0.547	0.531	0.355	0.340	0.578
OPT3	0.584	**0.823**	0.568	0.562	0.434	0.431	0.596
RESI1	0.546	0.433	**0.719**	0.386	0.118	0.159	0.410
RESI2	0.511	0.423	**0.725**	0.455	0.209	0.240	0.485
RESI3	0.662	0.610	**0.818**	0.637	0.409	0.389	0.578
RESI4	0.679	0.585	**0.805**	0.657	0.334	0.366	0.630
RESI5	0.524	0.429	**0.680**	0.517	0.286	0.309	0.479
SEEF1	0.630	0.526	0.636	**0.852**	0.417	0.461	0.742
SEEF2	0.450	0.488	0.495	**0.761**	0.340	0.355	0.580
SEEF3	0.564	0.508	0.575	**0.790**	0.407	0.464	0.698
SEEF4	0.500	0.457	0.540	**0.749**	0.406	0.381	0.585
SEEF5	0.520	0.386	0.473	**0.708**	0.299	0.345	0.548
SEEF6	0.552	0.534	0.545	**0.694**	0.262	0.290	0.632
SAT1	0.366	0.442	0.409	0.458	**0.850**	0.749	0.504
SAT2	0.277	0.285	0.252	0.345	**0.749**	0.594	0.355
SAT3	0.290	0.349	0.294	0.384	**0.805**	0.613	0.394
SAT4	0.267	0.367	0.294	0.399	**0.735**	0.568	0.399
SAT5	0.235	0.296	0.238	0.264	**0.831**	0.752	0.332
SAT6	0.322	0.347	0.287	0.390	**0.819**	0.748	0.467
ORGC1	0.351	0.380	0.320	0.420	0.781	**0.845**	0.465
ORGC2	0.256	0.325	0.271	0.344	0.704	**0.796**	0.414
ORGC3	0.311	0.374	0.332	0.397	0.734	**0.862**	0.489
ORGC4	0.305	0.334	0.266	0.334	0.497	**0.718**	0.402
ORGC5	0.324	0.384	0.342	0.452	0.624	**0.783**	0.466
ORGC6	0.402	0.371	0.391	0.490	0.707	**0.823**	0.549
INNO1	0.545	0.542	0.573	0.660	0.395	0.438	**0.795**
INNO2	0.626	0.477	0.572	0.638	0.318	0.376	**0.743**
INNO3	0.508	0.487	0.496	0.634	0.410	0.479	**0.742**
INNO4	0.501	0.502	0.512	0.579	0.392	0.470	**0.748**
INNO5	0.500	0.589	0.503	0.667	0.436	0.452	**0.814**
INNO6	0.494	0.486	0.460	0.552	0.400	0.407	**0.626**
INNO7	0.496	0.528	0.476	0.556	0.279	0.320	**0.646**
INNO8	0.496	0.426	0.438	0.565	0.228	0.286	**0.653**
INNO9	0.460	0.458	0.478	0.554	0.407	0.445	**0.687**

*The bolded values are standardized factor loadings for each measurement items, and the others are cross loadings. PSY, psychological capital; HOPE, psychological hope; RES, resilience; SEEF, self-efficacy; SAT, job satisfaction; ORGC, organizational commitment; INNO, employee innovative behavior.*

Discriminant validity mainly assesses the degree of difference between each construct in a measurement model. As shown in Table 5, based on the comparative results of the cross loading and factor loading of each indicator, the constructs of this study had good discriminant validity as the factor loading of each scale item of a specific potential construct was higher than the loading of any other construct (Hair et al., 2016).

### Structural Model

**FIGURE 2:**
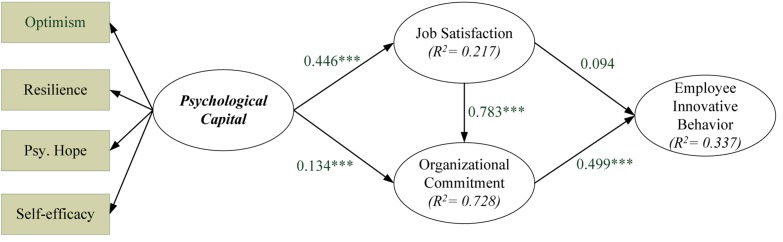
Path analysis result.

Structural model analysis was performed in this study after the analyses of reliability and construct validity. The PLS estimation results and structural model path coefficient obtained using PLS were used to determine the relationship between the constructs. The statistical hypothesis testing results are summarized in Figure 2 and Table 6.

**TABLE 6:** Summary of structural model results.

**Path direction**	**Standardized path coefficient**	***t*-value**	***p*-value**	**Result**
H1 (PSY → SAT)	0.466^∗∗∗^	6.030	0.000	Supported
H2 (PSY → ORGC)	0.134^∗∗∗^	3.724	0.000	Supported
H3 (SAT → ORGC)	0.783^∗∗∗^	26.617	0.000	Supported
H4 (SAT → INNO)	0.094	0.882	0.378	Not supported
H5 (ORGC → INNO)	0.499^∗∗∗^	5.256	0.000	Supported

*^∗∗∗^*p*-value < 0.001. PSY, psychological capital; HOPE, psychological hope; RES, resilience; SEEF, self-efficacy; SAT, job satisfaction; ORGC, organizational commitment; INNO, employee innovative behavior.*

### Mediation Analysis

The Sobel test and the path analysis approach were adopted to examine if the mediator variables in this study had any statistical significance. Prior to verifying the mediating effect, the predictive effect of the independent variable on the dependent variable, and the predictive effect of the independent variable on the mediator variable must be established. When the mediator variable is introduced, partial mediation occurs if the path coefficient between the independent variable and the dependent variable displayed correlation; whereas full mediation occurs if the path coefficient did not display correlation. Three testing methods were used to verify the mediating effect in this study (Aroian, 1947; Goodman, 1960; Sobel, 1982; MacKinnon et al., 1995), while the obtained *Z* scores were converted to *p*-values to determine if the mediating effect had any statistical significance (as shown in Table 7). Moreover, the percentile bootstrap method was used in this study to calculate the confidence intervals of the mediating effect. The mediating effect exists if the percentile bootstrap confidence intervals does not contain zero. Incidentally, the relevant mediating effects of a certain pathway were not analyzed if the pathway was not statistically significant (for example, SAT → INFO).

**TABLE 7:** Mediation effect examination.

**Mediation relationship**	**Path**	***t*-value of path**	**Sobel test’s *z*-value**	**Aroian test’s *z*-value**	**Goodman test’s *z*-value**
PSY → SAT → ORGC	PSY → SAT	6.030	5.880^∗∗∗^	5.877^∗∗∗^	5.885^∗∗∗^
	SAT → ORGC	26.617			
PSY → ORGC → INNO	PSY → ORGC	3.724	3.038^∗∗^	3.003^∗∗^	3.076^∗∗^
	ORGC → INNO	5.256			
SAT → ORGC → INNO	SAT → ORGC	26.617	5.156^∗∗∗^	5.153^∗∗∗^	5.160^∗∗∗^
	ORGC → INNO	5.256			

*^∗∗^*p*-value < 0.01; ^∗∗∗^*p*-value < 0.001.*

## Discussion and Conclusion

### Discussion

The PLS was adopted to estimate five hypotheses proposed in this study. Based on the entire samples, one path is not supported (H4), while the remaining paths are all significant at the 0.05 level (H1, H2, H3, and H5 are supported). Properties of the causal paths, including standardized path coefficients and hypotheses testing results in the hypothesized model are presented in Table 6.

Employee innovative behavior is predicted by organizational commitment (β = 0.499) and job satisfaction (β = 0.094), which jointly explained 29.5% of the variance in employee innovative behavior. Organizational commitment is influenced significantly by job satisfaction (β = 0.783) and PsyCap (β = 0.134), with jointly 78.3% of the total variance explained. Moreover, job satisfaction determined by PsyCap (β = 0.466), which jointly explain 19.7% of error variance on job satisfaction.

Although the impact of job satisfaction had no significant influence on employee innovative behavior in this study, we also estimated the serial mediation (i.e., PSY → SAT → ORGC → INNO) to prove the serial mediation effect (as shown in Table 7).

### Conclusion

Employees’ innovative behavior has received considerable interest in recent years, as governments and companies have begun to prioritize innovative developments. Innovation enables companies to operate sustainably in addition to enhancing their competitiveness. Innovation is not only important for research and development departments; rather, it is also essential for other departments. Employees themselves are the subjects of knowledge and innovation. Thus, organizations and companies should consider measures to promote and stimulate the innovative behavior of employees, so as to effectively enhance organizational knowledge and generate employees’ innovative behavior. In this study, the principles of positive psychology were applied in the field of positive organizational behavior to uncover the psychological strengths of individuals, and the PsyCap of individuals was proposed as an intrinsic mental resource that transcends economic capital, human capital, and social capital. Empirical studies have shown that an individual’s PsyCap is closely related to their work performance (Avey et al., 2011), positivity (Avey et al., 2008), and creativity (Rego et al., 2012). This indicates that individuals with high PsyCap are not only able to withstand challenges and changes and become successful employees, managers, or entrepreneurs, but are also capable of overcoming adversities and achieve greater organizational accomplishments.

Compared with large enterprises, small-and-medium enterprises (SMEs) have become the main carrier of China’s technological innovation and an important driving force for economic growth. In China, SMEs have become the mainstay of the Chinese economy because of their large number and scale. The innovative improvement of SMEs would enhance the industries transformation and industrialization of scientific and technological achievements, and the development of employment has shown remarkable development vitality. Based on the proposed model of this research, this paper explains how employees’ PsyCap affect organizational innovation behavior through organizational commitment and job satisfaction for SMEs. SMEs have unique advantages in flexible market adjustment and specialized production. However, due to their own characteristics, SMEs are facing more difficulties and challenges than ever before in the context of the slow recovery of the global economy including sensitivity to market fluctuations, weak ability to bear risks, etc. Thus, SMEs should consider to promote and stimulate the innovative behavior of employees, so as to effectively enhance organizational knowledge and generate employees’ innovative behavior.

Innovation-driven development has become China’s core national strategy. In 2015, China launched a major program of “Public Entrepreneurship and Innovation” to promote grassroots entrepreneurship across the country. This vision is also in line with China’s economic development goals, from labor-intensive manufacturing to innovation-led growth. China also has large-scale domestic consumer groups who are eager to acquire new technologies. Therefore, in the era of technological globalization, China companies have increasingly diverse demands for employees, which leads to the question: What attributes should workers have to stand out from the crowd? From a human resource perspective, PsyCap is an intangible asset of an individual that is not only related to work performance and job satisfaction, but also strengthens employees’ innovative behavior. Moreover, the results of this study have indicated that the PsyCap of organizational members was more able to promote their feedback inquiries and active participation in their jobs when there were no additional resources. Therefore, PsyCap can be used in practice as a reference indicator when recruiting employees, while the PsyCap of employees can be enhanced through their training process. In other words, the human resources management processes of selecting, training, and developing the PsyCap of organizational members are beneficial for improving their active participation in their jobs and enhancing the organizational efficiency.

Compared to current PsyCap-relevant studies which focus on organizational performance, future studies can investigate the moderating effects of different work scenarios. Additionally, by integrating the standpoints from the absence or presence of organizational resources, further studies can investigate the efficacy of an individual’s PsyCap when they were placed in advantageous or disadvantageous situations. For example, a recent study on leadership styles had stressed the effects of abusive supervision on subordinates’ organizational commitment, job satisfaction, and creative performance (Hoobler and Brass, 2006; Tepper, 2007; Liu et al., 2012). Hence, the role and efficacy of workers’ PsyCap on their expression of innovative behavior is a topic worth researching.

In this study, common-method variance may be present since self-reported data were concurrently collected from a common group of subjects. This bias was the main limitation of this study even though post-sampling statistical testing was performed to reduce it. For instance, the positive relationship between innovative efficacy and innovative behavior may be due to the employees’ self-reported responses. Furthermore, employees’ innovative behavior also includes personal innovation, which is neither easily observable nor understandable. Therefore, the use of employees’ self-reported innovative behavior can be considered appropriate for this study (Kaufman et al., 2009). Future studies can substantially reduce common-method variance by collecting employees’ innovative behavior data at different time points and utilizing data reported by managers and colleagues, or implementing other subjective methods.

## Data Availability Statement

All datasets generated for this study are included in the article/supplementary material.

## Author Contributions

YT conceived and designed the research, and wrote and revised the manuscript. Y-FS and Y-JC gave guidance throughout the whole research process.

## Conflict of Interest

The authors declare that the research was conducted in the absence of any commercial or financial relationships that could be construed as a potential conflict of interest.
